# Integrating mental health into primary health care in fragile and conflict-affected settings: a scoping review of mhGAP effectiveness

**DOI:** 10.1590/0102-311XEN199224

**Published:** 2025-10-03

**Authors:** Marcello Roriz de Queiroz, Elena Rubini, Martina Valente, Ives Hubloue, Francesco Della Corte

**Affiliations:** 1 Center for Research and Training in Disaster Medicine, Humanitarian Aid, and Global Health, Università del Piemonte Orientale, Novara, Italy.; 2 Vrije Universiteit Brussel, Brussels, Belgium.; 3 Department for Sustainable Development and Ecological Transition, Università del Piemonte Orientale, Novara, Italy.

**Keywords:** Mental Health, Primary Health Care, Capacity Building, Effectiveness, Emergency, Saúde Mental, Atenção Primária à Saúde, Fortalecimento Institucional, Efetividade, Emergência, Salud Mental, Atención Primaria de Salud, Creación de Capacidad, Efectividad, Emergencias

## Abstract

In 2008, the World Health Organization launched the Mental Health Gap Action Program (mhGAP) to scale up mental health care in non-specialized health care settings. Studies have demonstrated the benefits of mhGAP implementation while highlighting the need for better contextual adaptation and ongoing support. The challenge of integrating mental health into primary care is particularly noticeable in fragile and conflict-affected settings, where the need for such services is greater and health systems are often disrupted. A literature search was conducted on PubMed, PsycINFO, Scopus, and Web of Science to identify relevant peer-reviewed studies addressing the effectiveness of mhGAP in fragile and conflict-affected settings. Information was collected on study characteristics and design, impact of mhGAP, and main operational challenges. After full-text review, 10 articles met the inclusion criteria, reporting the impact of mhGAP on primary care personnel, on service user outcomes and in health systems. Studies reported post-training improvements in knowledge, mainly on epilepsy and psychosis. However, gaps remained in skills related to conducting mental state examinations, assessing suicide risk, and strengthening psychosocial support. The evidence was inconclusive regarding the impact of mhGAP on improving access to mental health care at the primary level. Several implementation challenges were identified, including an overemphasis on short-term knowledge transfer and the lack of structured supervision following mhGAP training.

## Abstract (pt)

Em 2008, a Organização Mundial da Saúde lançou o Programa de Ação para Lacuna em Saúde Mental (mhGAP, acrônimo em inglês) com o objetivo de ampliar o atendimento à saúde mental em serviços não especializados. Estudos demonstraram os benefícios do uso do mhGAP, ao mesmo tempo em que destacam a necessidade de melhor adaptação contextual e apoio contínuo. O desafio de integrar a saúde mental à atenção primária é particularmente acentuado em contextos precários e atingidos por conflitos, onde a necessidade desses serviços é maior e os sistemas de saúde estão muitas vezes interrompidos. Uma busca bibliográfica foi realizada nas bases de dados PubMed, PsycINFO, Scopus e Web of Science para identificar estudos relevantes que abordem a eficácia do uso do mhGAP em contextos precários e atingidos por conflitos. Foram coletadas informações sobre as principais características e o desenho dos estudos, o impacto do uso do mhGAP e os principais desafios operacionais. Após a análise dos artigos completos, 10 preencheram os critérios de inclusão, relatando o impacto do mhGAP nos profissionais de atenção primária, nos desfechos relacionados aos usuários e nos serviços de saúde. Os estudos relataram uma melhora do conhecimento após o treinamento, principalmente sobre epilepsia e psicose, enquanto lacunas nas habilidades foram relatadas na realização de um exame do estado mental, na avaliação do risco de suicídio e no fortalecimento do apoio psicossocial. Os estudos não apresentaram resultados conclusivos sobre o impacto do mhGAP para ampliar o acesso aos cuidados de saúde mental no nível primário de atenção. Vários desafios para implementar o mhGAP em contextos precários foram apresentados, inclusive o foco na transferência de conhecimento e a falta de supervisão estruturada após o treinamento do mhGAP.

## Abstract (es)

En 2008, se puso en marcha el Programa de Acción Mundial para Superar las Brechas en Salud Mental (mhGAP, por su sigla en inglés) de la Organización Mundial de la Salud, con el objetivo de reducir las brechas en salud mental y ampliar la atención de la salud mental en entornos de atención de salud no especializados. Los estudios han demostrado los beneficios del uso de mhGAP, al tiempo que destacan la necesidad de una mejor adaptación contextual y de un apoyo continuo. El desafío de integrar la salud mental en la atención primaria es particularmente pronunciado en entornos frágiles y afectados por conflictos, donde la necesidad de tales servicios es más aguda y los sistemas de salud se encuentran perturbados. Se realizó una búsqueda bibliográfica en PubMed, PsycINFO, Scopus y Web of Science para identificar estudios relevantes revisados por pares que abordaran la efectividad del uso de mhGAP en entornos frágiles y afectados por conflictos. Se recopiló información sobre las principales características y el diseño del estudio, el impacto del uso de mhGAP y los principales desafíos operativos. Después de la revisión del texto completo, 10 artículos cumplieron con los criterios de inclusión, informando sobre el impacto de mhGAP en el personal de atención primaria, en los resultados de los usuarios de los servicios y en los servicios de salud. Los estudios informaron una mejora de los conocimientos tras la formación, principalmente sobre la epilepsia y la psicosis, mientras que se identificaron lagunas en las habilidades para realizar un examen del estado mental, evaluar el riesgo de suicidio y fortalecer el apoyo psicosocial. Los estudios no mostraron resultados concluyentes sobre el impacto de mhGAP en la mejora del acceso a la atención de salud mental en el nivel primario de salud. Se identificó una serie de desafíos para implementar el mhGAP en entornos frágiles, incluidos el enfoque en la transferencia de conocimientos a corto plazo y la falta de supervisión estructurada después de la capacitación en mhGAP.

## Introduction

The integration of mental health into primary health care (PHC) represents a major shift in the global approach to address the growing need for mental health services ^1^. This shift is driven by the recognition that the mental health treatment gap remains a widespread challenge, especially in low-to-middle-income countries (LMICs). The World Health Organization (WHO) estimates that between 76% and 85% of people with severe mental disorders in these countries receive no treatment ^1^. Overall, LMICs have few mental health specialists in the public health sector, inpatient services for brief hospitalizations are scarce and limited to major cities, and medications are inconsistently available ^2^ ^,^ ^3^.

The importance of integrating mental health into PHC was firstly recognized during the 1978 *Alma-Ata Declaration* ^4^, which emphasized health as a fundamental human right and defined PHC as the cornerstone of health systems worldwide. The declaration called for the “*provision of basic preventive and curative mental health care at the first point of contact of entry into the health system*” ^4^ and laid the groundwork for early efforts to integrate mental health into PHC, highlighting its potential to reach underserved populations and improve overall health outcomes.

There is a broad consensus among health managers and global mental health experts on the relevance of integrating mental health into PHC. This approach is grounded on principles of accessibility, cost-effectiveness, and continuity of care. Integrating mental health services within PHC settings ensures availability at the community level, reducing distance and financial barriers that often prevent individuals from seeking help. It also supports continuous care, as PHC providers are typically the first point of contact within the health system and can address both physical and mental health needs comprehensively ^5^ ^,^ ^6^ ^,^ ^7^. However, several barriers - such as stigma, lack of trained personnel, inadequate funding, and fragmented health systems - continue to affect the pathways to care ^8^.

As part of global efforts to address the large burden of mental health conditions, national health authorities and international organizations led by the WHO have implemented initiatives to scale up care for mental health and substance use disorders at the PHC level. In 2008, the WHO launched the Mental Health Gap Action Program (mhGAP) to expand care for mental, neurological, and substance use (MNS) disorders in non-specialized health care settings. The implementation of mhGAP includes a series of manuals and training tools (mhGAP-IG) designed to equip PHC practitioners with the skills and knowledge required to manage common mental health conditions ^9^. A revised version, mhGAP-IG 2.0, was released in 2015 ^10^. Studies have demonstrated that mhGAP-IG training improves health workers’ knowledge, attitudes, and confidence in managing mental health disorders ^11^, while also highlighting the need for better contextual adaptation and ongoing support to sustain these gains ^6^.

The challenge of integrating mental health care into PHC settings is particularly evident in humanitarian crises, in which the need for such services is most urgent. These crises, often triggered or exacerbated by armed conflicts, create extreme hardship environments, where access to basic necessities is severely limited. In such settings, the risk of traumatic exposure increases, and the prevalence and burden of common mental disorders are significantly higher than global estimates ^12^ ^,^ ^13^. Additionally, health systems are often damaged, medication supplies disrupted, and material resources scarce ^14^. Considering this scenario, integrating mental health care into PHC is not only challenging but essential. In response to these challenges, the WHO, in collaboration with the United Nations High Commissioner for Refugees (UNHCR), developed the mhGAP Humanitarian Intervention Guide (HIG), an adapted version with specific conceptualization, assessment principles, and adapted modules, launched in 2015 ^15^.

By conducting an extensive review of gray literature, Spagnolo & Lal ^16^ identified over 90 countries where the mhGAP-IG was implemented. A systematic review by Keynejad et al. ^17^ also reported a growing number of studies on the use and evaluation of mhGAP-IG in recent years, mostly conducted in LMICs. However, limited understanding of mhGAP use in fragile settings and a shortage of evidence on its effectiveness limit the analysis of operational and technical challenges, as well as potential strategies to improve its implementation. To our knowledge, this is the first review focusing on the effectiveness of mhGAP use in fragile and conflict-affected settings.

The purpose of this scoping review is to summarize evidence from peer-reviewed studies on the effectiveness of the implementation of the WHO mhGAP-IG in conflict-affected, fragile, and emergency settings. Specifically, it aims to analyze the effectiveness of mhGAP-IG training packages on PHC personnel, service users, and health services, as well as to document the challenges and recommendations identified in the studies.

## Methods

A scoping review of published scientific peer-reviewed articles on the effectiveness of mhGAP-IG in conflict-affected and emergency settings was conducted according to the Joanna Briggs Institute (JBI) manual for evidence synthesis guidelines and was reported following the *Preferred Reporting Items for Systematic Reviews and Meta-Analyses: Extensionj for Scoping Reviews* (PRISMA-ScR) ^18^ ^,^ ^19^ ^,^ ^20^. Research team members have a background in global health, mental health, and humanitarian aid.

### Data sources and search strategy

A comprehensive search was conducted on August 30, 2024, in four databases (PubMed, PsycINFO, Scopus, and Web of Science) to identify all relevant peer-reviewed studies reporting on the effectiveness of mhGAP-IG in fragile and conflict-affected settings. An additional manual search was conducted on Google Scholar to identify other eligible studies. To define the scope of fragile and conflict-affected settings in any given year, we followed the annual World Bank classification of fragile and conflict-affected settings, which also includes post-conflict contexts ^21^. The search strings combined two sets of terms: one related to mhGAP, and the other to fragile and conflict-affected settings (Supplementary Material - Box S1; https://cadernos.ensp.fiocruz.br/static//arquivo/suppl-e00199224_8019.pdf). As the WHO mhGAP was launched in 2008 and the World Bank classification started in 2009, we included articles published between January 1, 2009 and August 30, 2024.

### Inclusion/exclusion criteria

Studies were included if they met the following inclusion criteria: (a) they were original studies reporting on the implementation of mhGAP-IG; (b) they were published between January 1, 2009 and August 30, 2024; and (c) they reported any dimension of impact or relevant discussion related to mhGAP implementation. Studies were included if they were conducted in fragile or conflict-affected contexts, according to the World Bank classification, or if they described mental health responses in emergency settings ^21^. Studies focusing on services for refugee populations were also included, regardless of whether the host country was listed in the World Bank classification in the year of implementation. For studies conducted in multiple countries, inclusion was based on whether at least one country was classified as fragile or conflict-affected. Considering the wide adaptation of mhGAP by national authorities and humanitarian agencies, we included any study that reported the use of at least two mhGAP-IG modules. No exclusion criterion was applied to studies published in languages other than English. Studies were excluded if they did not meet the inclusion criteria, if they were literature reviews, or if they only presented adapted mhGAP materials, training curricula, or research protocols.

### Data extraction and analysis

The results from the selected databases were stored and combined using the Zotero software, version 7.0 (https://www.zotero.org/). After duplicate removal and title and abstract screening, articles eligible for full-text review were organized in a Google Spreadsheet (https://spreadsheets.google.com/) for data extraction (Supplementary Material - Box S2; https://cadernos.ensp.fiocruz.br/static//arquivo/suppl-e00199224_8019.pdf). Data included general information about the article, study design, and details on mhGAP implementation and effectiveness, as well as challenges, gaps, and recommendations identified by the authors.

## Results

**Figure 1:**
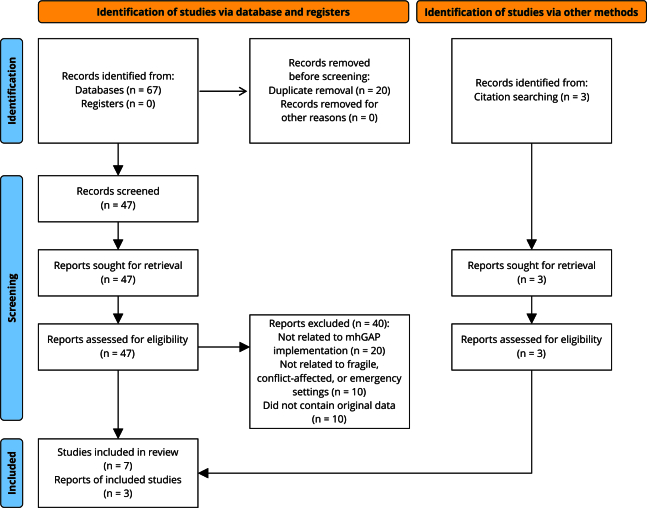
Study selection process.

The search yielded 70 articles. After removing duplicates and screening title and abstracts, 47 articles were eligible for full-text review. Of these, 10 met the inclusion criteria. Detailed information regarding the screening process can be found in the PRISMA diagram (Figure 1) ^18^. Box 1 shows a comprehensive overview of the main study characteristics.

mhGAP: Mental Health Gap Action Program.

**Box 1:** Characteristics of the included studies.

STUDY	COUNTRY	STUDY PERIOD	STUDY TYPE	STUDY OBJECTIVE	POPULATION
Echeverri et al. ^22^	Cameroon, Chad, Democratic Republic of the Congo, Ethiopia, Kenya, Uganda, and Tanzania	2015-2017	Qualitative	Describes and analyzes how the capacity building program was perceived among trainees and other local stakeholders, evaluating its effects	54 clinicians and 30 community workers from 6 countries that participated in the mhGAP training
Tarannum et al. ^24^	Bangladesh	2017	Mixed methods	Describes how UNHCR and health partners worked towards the integration of mental health into primary care	62 primary health care workers involved in the mhGAP training. Most (36) were physicians. Others were medical assistants (10), psychologists (6), health educators (7), medical coordinators (2), or nurses (1)
Siriwardhana et al. ^25^	Sri Lanka	2014	Mixed methods	Tests the feasibility of improving identification, treatment, and referral of common mental disorders by primary care practitioners using mhGAP-based training. The secondary objective was to explore attitudes and perceptions of primary care practitioners on integrating mental health into primary care	12 primary care practitioners from the Northern Province of Sri Lanka
Hughes et al. ^29^	Iraq, Turkey, and Syria	2014-2016	Qualitative	Aims to review a training program in Iraq, Turkey, and Syria, whose goal is to improve access to mental health care for affected vulnerable populations	98 health care providers from Syria (30), Iraq (52), and Turkey (16)
Humayun et al. ^26^	Pakistan	2014	Quantitative	Describes the training process including the adaptation of the mhGAP curriculum, training of trainers, training workshops for primary care staff, and analysis of results of pre- and post-testing of their knowledge about common mental disorders using a 25-item questionnaire	58 participants, including physicians (51) and psychosocial staff of humanitarian agencies (7)
Kohrt et al. ^23^	Uganda, Liberia, and Nepal	2015	Quantitative	Evaluates competency among primary care workers trained in mhGAP and community-based mental health services for a program targeting psychosis (including mania) and epilepsy in settings impacted by humanitarian conflict	206 health workers in humanitarian settings
Momotaz et al. ^27^	Bangladesh	2017-2018	Qualitative	Describes the adaptation of mhGAP training guidelines, results assessment, and practical challenges encountered during implementation	21 participants, including physicians from government facilities (8), humanitarian agencies working in the refugee camps (7), and psychosocial staff from humanitarian agencies (6)
Agudelo-Hernández et al. ^31^	Colombia	2023	Mixed methods	Describes barriers and challenges on mhGAP implementation and determines the correlation between facilitators, accessibility, acceptance, and the component of supervision	41 participants: 30 health personnel (5 physicians, 7 nurses, 18 psychologists) and 11 administrative workers
Doherty et al. ^28^	Sri Lanka	2018-2021	Quantitative	Measures stigma using pre- and post-mental health knowledge training to understand if stigma among healthcare professionals and community members could be reduced with knowledge gain	22 primary care practitioners, 61 public health professionals across 22 primary care facilities, and 48 community representatives from facility catchment areas
Al-Uzri et al. ^30^	Iraq	2022	Quantitative	Assesses the impact of mhGAP-IG 2.0 training in MNS disorders in improving physicians’ knowledge of evaluation and management of MNS conditions	17 primary care physicians

mhGAP: Mental Health Gap Action Program; MNS: mental, neurological and substance use disorders; UNHCR: United Nations High Commissioner for Refugees.

### Characteristics of the included studies

**Figure 2:**
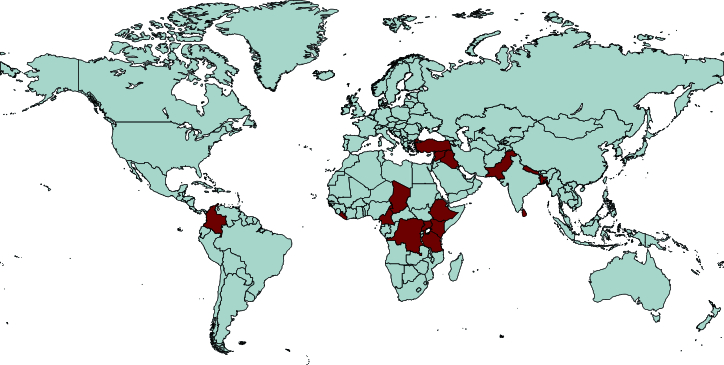
Countries where the studies were conducted.

Overall, the included studies reported the implementation of mhGAP in sub-Saharan Africa (Cameroon, Chad, the Democratic Republic of Congo, Ethiopia, Kenya, Uganda, Tanzania, and Liberia) ^22^ ^,^ ^23^, South Asia (Bangladesh, Nepal, Pakistan, and Sri Lanka) ^23^ ^,^ ^24^ ^,^ ^25^ ^,^ ^26^ ^,^ ^27^ ^,^ ^28^, the Middle East (Iraq, Turkey, and Syria) ^29^ ^,^ ^30^, and South America (Colombia) ^31^. Figure 2 shows a map of countries where the studies were conducted.

Eligible studies adopted the following methodologies: quantitative (pre-post designs and randomized controlled trials [RCT], n = 4) ^23^ ^,^ ^26^ ^,^ ^28^ ^,^ ^30^, mixed methods (n = 3) ^24^ ^,^ ^25^ ^,^ ^31^, and qualitative studies (n = 3) ^22^ ^,^ ^27^ ^,^ ^29^. Most participants involved in mhGAP programs were PHC practitioners (57.8%, n = 422); others were community health workers (4.1%, n = 30), psychologists (4.1%, n = 30), other physicians (1.6%, n = 12), health managers, and staff from local nongovernmental (NGOs) and humanitarian organizations.

The studies reported mhGAP implementation in countries classified as fragile and conflict-affected by the World Bank classification (the Democratic Republic of Congo, Chad, Iraq) ^22^ ^,^ ^29^ ^,^ ^30^, in post-conflict settings (Sri Lanka, Uganda, Liberia, and Nepal) ^22^ ^,^ ^23^ ^,^ ^25^ ^,^ ^28^, in contexts focused on services for refugee or internally displaced populations (Myanmar, Syria, Pakistan) ^24^ ^,^ ^26^ ^,^ ^27^ ^,^ ^29^, or in municipalities that declared humanitarian emergency (Colombia) ^31^.

Overall, eligible studies aimed to describe and analyze the implementation of mhGAP-IG, addressing a wide range of aspects, such trainees’ and stakeholders’ perceptions of mhGAP capacity building ^22^ ^,^ ^25^, acquired knowledge and skills ^23^ ^,^ ^25^ ^,^ ^26^ ^,^ ^29^ ^,^ ^30^, perceived changes in PHC providers’ attitudes towards mental health care ^24^ ^,^ ^28^, and barriers and challenges encountered during mhGAP implementation ^27^ ^,^ ^31^.

### Effectiveness of mhGAP

All eligible studies reported on the effectiveness of mhGAP implementation in at least one dimension of impact (on PHC personnel, service user outcomes, or health services and policies). Nine out of the 10 studies reported outcomes related to PHC personnel, while two addressed service user outcomes and two focused on health services and policies ^31^.

#### Impact on PHC personnel

Most studies reporting the impact of mhGAP on PHC personnel used pre-post knowledge tests adapted from the questionnaire proposed in the mhGAP guidelines ^24^ ^,^ ^25^ ^,^ ^26^ ^,^ ^29^ ^,^ ^30^. These studies reported improvements in acquired knowledge ranging from 6% to 25%. Overall, the main knowledge improvements were reported on content related to epilepsy, psychosis, and common misconceptions about the addictive potential of antidepressants and benzodiazepines ^23^ ^,^ ^26^ ^,^ ^27^. Smaller improvements were reported in knowledge related to suicide ^23^ ^,^ ^26^.

Some studies used training reports, in-depth interviews, supervisor observation, and field monitoring reports to assess acquired skills and changes in attitudes among PHC personnel ^22^ ^,^ ^24^ ^,^ ^25^ ^,^ ^27^. Acquired skills were reported in communication with service users and on quality of psychotropic prescriptions ^22^ ^,^ ^27^. One qualitative study reported self-perceived improvement in knowledge of common mental health conditions, basic case assessment skills, and care management in practical situations ^22^.

Main skill gaps were reported in conducting mental state examinations, assessing suicide risk, providing psychoeducation, and strengthening psychosocial support - including the involvement of family members in care ^23^ ^,^ ^24^ ^,^ ^26^ ^,^ ^27^. One RCT study reported no meaningful reduction in stigma towards mental health among trained PHC personnel ^28^.

#### Service user’s outcome

In the two studies reporting outcomes for service users, patients were either not systematically assessed or only a subset of the studied population was evaluated ^23^ ^,^ ^24^. Both studies reported evidence of significant improvement among individuals with epilepsy and psychotic disorders, including a reduced number of seizures, decreased severity of psychotic symptoms, and improved social functioning. In both patient groups (e.g., epilepsy and psychotic disorders), reductions were also observed in depressive symptoms, functional disability, and family and caregiver burden ^23^.

#### Health service and policy

Findings on the impact of mhGAP implementation on the increase of patients benefiting from mental health care at the primary level were inconclusive according to the two studies that assessed this dimension ^22^ ^,^ ^24^. In one study, the number of consultations for MNS conditions in trained health centers increased fourfold ^22^. In a multi-center study, the numbers of monthly consultations for some MNS conditions increased after training, whereas for others the numbers either decreased, remained stable, or fluctuated over time ^22^.

### Challenges to operate in fragile contexts

This review identified several dimensions of challenges faced by national health authorities and implementing partners to conduct the mhGAP-IG in fragile and conflict-affected settings. One major obstacle was the high turnover of PHC staff during and after the training process ^22^ ^,^ ^24^, which led to losing the training investment and to a lack of continuity in care. This turnover arose from insecure employment conditions and the difficult environments in which PHC staff were operating ^24^. The scarcity of mental health specialists also created barriers to effective referrals and continuity of care ^22^. The situation became particularly dire during periods of intensified conflict, when professionals were forced to flee, further reducing service availability.

A second major challenge was ensuring ongoing training and professional development. PHC providers working in conflict zones often lacked access to education and regular supervision ^30^ ^,^ ^31^. Participation in training programs, including mhGAP initiatives, was hindered by insecurity and high patient demands ^27^. This lack of continuous education left healthcare workers feeling unprepared to address complex mental health issues.

Logistical challenges, such as poor internet connection and budget constraints, also impacted the effectiveness of mhGAP interventions ^24^ ^,^ ^31^. In many settings, there was a shortage of resources to support these programs, leading to underfunded and understaffed services ^10^.

Regarding workload, PHC practitioners in conflict-affected zones often faced overwhelming patient numbers, limiting their ability to properly identify and treat mental health disorders ^25^ ^,^ ^27^. Furthermore, the lack of community-based services and limited referral pathways hindered the provision of adequate care - particularly in refugee camp settings, where services are especially scarce ^26^ ^,^ ^27^ ^,^ ^30^. In these settings, refugee workers were invaluable resources for healthcare delivery ^29^.

Another significant obstacle was the lack of leadership from local authorities. Mental health is frequently deprioritized in fragile settings, resulting in little support for its integration into PHC ^26^. Finally, mental health stigma remained a considerable barrier. Stigmatization discouraged individuals from seeking treatment and contributed to discrimination within healthcare settings, further complicating efforts to integrate mental health into PHC ^28^.

### Challenges identified on the design and implementation of mhGAP

Limitations in mhGAP implementation that hindered its effectiveness were observed across eligible studies. One key issue was the insufficient involvement of governmental stakeholders when mhGAP initiatives were led by partner organizations, which often resulted in limited national ownership and reduced sustainability. This could be reflected in the lack of motivation among trainees at the beginning of the capacity-building process ^22^ ^,^ ^25^.

Most studies reported the mhGAP curriculum was adapted and condensed into 3 to 5 days of training. This brief training was described as insufficient to equip PHC workers with the necessary skills and was associated with minimal skill improvements as measured by the pre-post knowledge tests ^23^ ^,^ ^24^ ^,^ ^27^ ^,^ ^30^.

Another limitation was the lack of content on certain topics (e.g., child- and family-focused mental health and psychosocial support [MHPSS] approaches) and the need for better contextualization of mental health care in conflict-affected settings ^24^ ^,^ ^25^. Furthermore, inadequate selection of trainees contributed to inconsistencies in training delivery and outcomes ^22^. The efficacy of training videos was also limited by language barriers ^25^. Ideally, the mhGAP curriculum should be codeveloped with local stakeholders, and a proper training-of-trainers model should be implemented to ensure better outcomes ^27^.

Despite the consensus among studies that regular supervision and post-training follow-up are crucial for mhGAP’s effectiveness, the lack of standardized supervision protocols was identified as a limitation ^23^. In this sense, the shortage of mental health specialists, especially psychiatrists, to supervise trained staff emerged as a major challenge ^26^ ^,^ ^31^.

Some studies reported that practitioners, particularly physicians, perceived the clinical supervision model as a threat to their autonomy and were often hesitant to engage in psychosocial approaches, fearing these interventions would be too time-consuming or that they were outside their expertise ^24^ ^,^ ^26^. This attitude could be connected to the fact that mental healthcare in many countries remains limited to the prescription of psychiatric medications ^26^.

Moreover, the studies highlighted that the standardized mhGAP model often requires adaptation to the local sociocultural context ^25^ ^,^ ^30^. Understanding cultural norms and perceptions towards mental health was deemed critical, especially in refugee settings, in which specific cultural aspects directly impacted health service delivery and use ^27^.

## Discussion and recommendations

Despite the widespread implementation of the mhGAP-IG in LMICs and its relevance in mental health responses during humanitarian crises, peer-reviewed literature on its effectiveness remains limited. This gap highlights a crucial need for better documentation of mhGAP use, including critical discussions about its dimensions of impact. In fragile and conflict-affected settings, in which mental health needs are particularly acute, robust evidence is required to better understand mhGAP’s role in improving access to quality mental health care at the PHC level. Studies emphasizing qualitative aspects of implementation, incorporating perspectives from professionals and service users, could enhance understanding of how mhGAP can be adapted and improved in different contexts.

### Effectiveness of mhGAP in improving access to quality mental health care

When evaluating the effectiveness of mhGAP in improving access to mental health care at the PHC level, the studies yielded inconclusive results. Few studies have assessed mhGAP’s impact on service accessibility, and among these, the reported data is insufficient to confirm a direct link between mhGAP training and improved access to care. The two studies that evaluated monthly consultations for MNS conditions after mhGAP training did not reach a conclusion on the increase of service delivery post-training ^22^ ^,^ ^24^. The limited evidence on service user outcomes also constrains the capacity to determine whether mhGAP training has a tangible impact on the quality of mental health care provided by trained PHC personnel ^26^ ^,^ ^32^ ^,^ ^33^ ^,^ ^34^.

### Challenges in coordinating a comprehensive strategy in fragile and conflict-affected settings

The implementation of mhGAP in conflict, post-conflict, and other humanitarian crises settings have additional challenges due to the inherent instability of these contexts. These settings pose a wide range of limitations that undermine the sustainability of mhGAP at multiple levels - including high staff turnover, limited resources, and insufficient leadership from local authorities. Many governments remain underprepared and under-resourced to support the integration of mental health services into PHC, resulting in a heavy reliance on international organizations for implementation. This reliance often leads to fragmented service delivery and a lack of coordination among stakeholders, further complicating efforts to ensure sustainability ^35^ ^,^ ^36^ ^,^ ^37^.

### Focus on knowledge transfer and skills gaps

Most eligible studies focused on pre-post knowledge assessments, reflecting an emphasis on improving the knowledge base of PHC practitioners. Furthermore, knowledge-level assessments are easier to conduct compared to evaluating mhGAP’s effectiveness in improving access to care, which requires more complex monitoring mechanisms, including adapted tools and follow-up. While studies reported knowledge gains - mainly on epilepsy, psychosis, and prescription of psychotropic medications ^23^ ^,^ ^26^ ^,^ ^27^ - the focus on acquired knowledge does not necessarily translate into improved service delivery or greater access to care ^23^ ^,^ ^38^. This narrow focus on knowledge transfer also underscores the limitations of brief, short-term training models and operational challenges, including the lack of comprehensive strategies to sustain the impact of mhGAP in fragile and conflict-affected settings.

While mhGAP training has shown promising results in improving knowledge and skills related to physician-driven contents (e.g., psychotropic prescription practices and management of epilepsy and psychosis), the studies identified gaps in psychoeducation and strengthening psychosocial support. The excessive focus on knowledge transfer of physician-driven contents may risk overlooking cultural factors within the clinical encounter. Understanding local expressions of emotional suffering, often referred to as “idioms of distress,” is crucial for the effective implementation of mhGAP-IG ^39^. These culturally specific ways of communicating psychological pain shape how individuals recognize, interpret, and seek help for mental health issues ^40^. If such expressions are overlooked, health workers may misidentify or fail to detect common mental disorders, leading to underdiagnosis and ineffective care.

The reported hesitance from PHC staff to engage in psychosocial support is often connected to a traditional psychiatrist-centered mindset in mental health care, which remains heavily reliant on biomedical approaches ^41^. This highlights the persistent gap between biomedical and psychosocial models of care. Additionally, other skill gaps were reported in conducting mental state examinations and assessing suicide risk - skills that normally require more extensive training and supervised clinical practices.

### **“*Capacity building is a process and not an event*”** ^24^ **(p. 137)**

Most studies reported the use of an adapted mhGAP curriculum, condensed into 3 to 5 days of training. These abbreviated training models, widely implemented in LMICs, present significant limitations in achieving its goal of improving access to quality mental health care at the PHC level. This condensed format offers only surface-level exposure to complex mental health issues and, while PHC practitioners may have some knowledge improvement of key topics, significant gaps remain in handling cases, psychosocial interventions, and broader mental health care strategies ^6^. Essential skills require more extensive training and hands-on experience, which cannot be adequately addressed with such short training formats ^23^ ^,^ ^24^ ^,^ ^27^.

Another significant gap reported by the studies regards the lack of structured supervision following mhGAP training. Studies that included post-training supervision noted improvements in healthcare providers’ communication skills, empathy, and capacities for multi-level care management ^22^ ^,^ ^27^. However, in most reported experiences, ongoing supervision was either insufficient or absent, limiting knowledge retention and the practical application of acquired skills. Without structured follow-up, PHC staff often revert to previous practices, especially in challenging environments where mental health is stigmatized or deprioritized ^42^. Finally, short-term training alone is insufficient to address systemic issues inherent in fragile and conflict-affected settings, where staff turnover is high and mental health services are rarely integrated into the health system. A more sustained and comprehensive training approach, complemented by ongoing supervision, is necessary to achieve long-term improvements in mental health service delivery at the PHC level ^10^.

### Monitoring framework in mhGAP implementation

There is a growing need for the use of a systematic framework to monitor mhGAP implementation, especially during emergency responses. This framework should involve routine collection of data on essential indicators to monitor patient trends and progress over time. Monitoring efforts should go beyond assessing knowledge and skills, encompassing patient outcomes, service delivery improvements, and the overall sustainability of the program. In conflict-affected settings, where the long-term impact of mhGAP is often difficult to sustain, such a framework could help identify implementation gaps and guide strategies to enhance the program’s effectiveness.

### Integration of mhGAP into pre-service training

The core objective of mhGAP-IG - improving the mental health knowledge of healthcare workers - enormously benefits when including adapted mhGAP-based curricula into formal medical education programs ^43^. A crucial area for improvement is the integration of mhGAP into pre-service training ^44^. Reinforcing mental health knowledge and skills during medical education could ensure that PHC practitioners are better prepared to address mental health conditions throughout their careers. Incorporating mhGAP modules in pre-service curricula would also help bridge the gap between biomedical and psychosocial approaches, improving the capacity of healthcare workers to manage mental health conditions, particularly in complex and resource-limited settings.

### Addressing stigma

The pervasive stigma surrounding mental health remains a significant barrier. This stigma exists not only within communities but also among healthcare workers, who may have internalized societal norms toward individuals with mental disorders. To overcome this barrier, mhGAP training must include more practical modules focused on addressing stigma, using approaches adapted to local cultural norms. Additionally, greater investment in community-based interventions is needed to raise awareness about mental health issues and reduce stigma at the grassroots level.

### Limitations

This review has some limitations. First, few studies assessed the impact of mhGAP on service accessibility, and the reported data are insufficient to confirm a direct link between mhGAP training and improved access to care. Furthermore, only two studies reported outcomes at the service user level, limiting the understanding of mhGAP-IG effectiveness. Moreover, due to the search strategy employed, gray literature was excluded.

## Conclusion

The mhGAP-IG has been widely used, including as a key training tool in humanitarian crises contexts. However, its effectiveness remains under-evaluated in the peer-reviewed literature. Most existing studies focus on acquired knowledge among PHC providers, yet offer limited evidence linking mhGAP training to improved access to mental health care at the primary care level. Limited monitoring on service user outcomes constrains the ability to determine whether mhGAP has led to tangible improvements in the quality of care provided by trained PHC staff in fragile and conflict-affected contexts.

The emphasis on short-term knowledge transfer, combined with the lack of structured post-training supervision, limits the potential of mhGAP on improving access to quality mental health care at the PHC level. Operational and coordination issues further undermine the sustainability of mhGAP initiatives, especially in settings where local health systems are weak or fragmented. To maximize the impact of mhGAP, operational and strategic measures are required, including a more sustained and comprehensive training approach, stronger local leadership, improved coordination mechanisms, and the implementation of an efficient and adaptable monitoring framework to assess mhGAP effectiveness and ensure the long-term integration of mental health care into primary health systems.
