# Supplementary material for: Integrating mental health into primary health care in fragile and conflict-affected settings: a scoping review of mhGAP effectiveness
Source: Cad Saude Publica. 2025 Oct 3;41(9):e00199224. doi: 10.1590/0102-311XEN199224 (PMC12494388; doi:10.1590/0102-311XEN199224)
Supplement: Supplementary file 1 [file 1678-4464-csp-41-09-EN199224-s.pdf]

SUPPLEMENTARY MATERIAL

Box S1 Search strings.

DATABASE	SEARCH STRINGS	OUTCOMES
MEDLINE/PubMed (30/Aug/2024)	(TITLE-ABS-KEY (conflict) OR TITLE-ABS-KEY (disaster) OR TITLE-ABS-KEY (humanitarian) OR TITLE-ABS-KEY (emergency) OR TITLE-ABS-KEY (war) AND TITLE-ABS-KEY (mhGAP-IG) OR TITLE-ABS-KEY (mhGAP)	23
Scopus (30/Aug/2024)	(TITLE-ABS-KEY (conflict) OR TITLE-ABS-KEY (disaster) OR TITLE-ABS-KEY (humanitarian) OR TITLE-ABS-KEY (emergency) OR TITLE-ABS-KEY (war) AND TITLE-ABS-KEY (mhGAP-IG) OR TITLE-ABS-KEY (mhGAP)	24
PsychInfo (30/Aug/2024)	(TITLE-ABS-KEY (conflict) OR TITLE-ABS-KEY (disaster) OR TITLE-ABS-KEY (humanitarian) OR TITLE-ABS-KEY (emergency) OR TITLE-ABS-KEY (war) AND TITLE-ABS-KEY (mhGAP-IG) OR TITLE-ABS-KEY (mhGAP)	0
Web of Science (30/Aug/2024)	(TITLE-ABS-KEY (conflict) OR TITLE-ABS-KEY (disaster) OR TITLE-ABS-KEY (humanitarian) OR TITLE-ABS-KEY (emergency) OR TITLE-ABS-KEY (war) AND TITLE-ABS-KEY (mhGAP-IG) OR TITLE-ABS-KEY (mhGAP)	20
**TOTAL**		67

Box S2 Extraction sheet.

**General information of the article**	Title
	First author
	Publication year
	Language
	Country where the study was conducted
**Study design**	Study period
	Objective of the study
	Study type
	Methodology
	Population
**Impact of implementation of mhGAP (evidence and measures applied)**	On PHC personnel
	On service users outcomes
	On health services
	On health policies
**Setting**	Conflict-affected
	Fragile
**Recommendations**	Recommendations from participants and authors
**Limitations**	Study limitations (authors of the studies)
	Study limitations (authors of the review)
**Considerations**	Notes
